# A Hybrid Rule-Based and Machine Learning–Based Clinical Decision Support System to Support Prescription Review: Development and External Validation Study

**DOI:** 10.2196/101202

**Published:** 2026-09-23

**Authors:** Jonghyun Jeong, Kyu-Nam Heo, A Jeong Kim, Jin Hee Baek, Yun Hee Jo, Sunghwan Kim, Mingi Jo, Young-Mi Ah, Ji Min Han, Sae Won Choi, Junho Song, Sangil Min, Ju-Yeun Lee

**Affiliations:** 1 College of Pharmacy, Seoul National University, 1 Gwanak-ro, Gwanak-gu, Seoul, 08826, Republic of Korea, 82-2-3668-7472; 2 College of Pharmacy and Research Institute of Pharmaceutical Sciences, Seoul National University, Seoul, Republic of Korea; 3 Department of Pharmacy, Seoul National University Hospital, Seoul, Republic of Korea; 4 ZerOne AI Inc, Seoul, Republic of Korea; 5 College of Pharmacy, Yeungnam University, Daegu, Republic of Korea; 6 College of Pharmacy, Chungbuk National University, Cheongju, Republic of Korea; 7 Office of Hospital Information, Seoul National University Hospital, Seoul, Republic of Korea; 8 Department of Surgery, Seoul National University Hospital, Seoul, Republic of Korea

**Keywords:** clinical decision support system, medication errors, anticoagulants, machine learning, electronic health records, patient safety

## Abstract

**Background:**

Anticoagulants are high-alert medications with substantial risk of serious bleeding, yet dosing and prescribing errors remain common. Although clinical decision support systems (CDSSs) can mitigate these errors, their impact is constrained by alert fatigue and limited interpretability.

**Objective:**

We developed and validated a hybrid CDSS that integrates rule-based logic with machine learning to improve the safe use of anticoagulants.

**Methods:**

This multicenter study used electronic health record data on anticoagulant prescriptions from 3 tertiary hospitals (1 for system development and internal validation and 2 for external validation). The hybrid CDSS combined a knowledge-based rule engine with a machine learning model trained to predict whether an anticoagulant prescription would require pharmacist intervention. The system was iteratively refined through pilot testing, internal validation, and external validation.

**Results:**

A total of 75,200 anticoagulant prescriptions were used for model development. The final hybrid CDSS comprised 44 patient-specific rules and 1129 drug-drug interaction rules, combined with a CatBoost classifier (version 1.2.5; Yandex) for alert prioritization. During internal validation, 88 (18.9%) alerts were generated; all were technically correct; 88.6% (n=78) were deemed clinically relevant; 86.4% (n=76) were considered clinically useful; and 13.6% (n=12) required pharmacist intervention. In external validation across 2 hospitals, alert rates ranged from 22.6% (7/31) to 32.1% (310/966), with 18.8% (22/117) to 57.1% (4/7) of alerts requiring pharmacist intervention. The hybrid CDSS showed strong discrimination (area under the receiver operating characteristic curve 0.871‐0.963). No false negatives were identified, but the estimates should be interpreted cautiously given the short validation periods and limited number of intervention-requiring prescriptions.

**Conclusions:**

A hybrid CDSS integrating rule-based logic with machine learning demonstrated high technical accuracy and clinical relevance across multiple institutions, suggesting its potential as a practical tool for supporting pharmacist-led anticoagulant prescription review.

## Introduction

### Background

Anticoagulants are cornerstone therapies for stroke prevention in atrial fibrillation and for the treatment and prophylaxis of venous thromboembolism (VTE). However, their complex dosing criteria and monitoring requirements across oral and injectable anticoagulants make them susceptible to medication errors. Warfarin therapy is complicated by numerous drug and food interactions, necessitating regular international normalized ratio (INR) monitoring. Although direct oral anticoagulants (DOACs) have established themselves as the mainstay of anticoagulant therapy owing to their superior safety and convenience over warfarin, precise dose selection based on age, body weight, renal function, and other clinical factors remains essential [1]. Accordingly, prescribing errors remain frequent; a recent systematic review reported that approximately 20% of DOAC prescriptions contain at least one error [2]. Because inappropriate anticoagulant use can lead to serious harm, anticoagulants are classified as high-alert medications in international safety frameworks and in Korea [3-6]. Pharmacovigilance data further indicate that a substantial proportion of bleeding events associated with oral anticoagulants was preventable, mainly due to drug-drug interactions or inappropriate dosing [7].

Medication errors involving anticoagulants are not limited to overdose-related bleeding; underdosing with subsequent thromboembolism is also a critical concern. Off-label DOAC use in patients with atrial fibrillation has been reported in 24% of cases overall, with higher prevalence in Asia (32%) compared with North America (14%) or Europe (22%) [8]. This pattern may reflect heightened concern about bleeding risk in Asian practice settings [9]. However, meta-analyses have shown that off-label underdosing is associated with increased risks of ischemic stroke or systemic embolism without a corresponding reduction in major bleeding, whereas off-label overdosing is associated with higher risks of both major bleeding and thromboembolic events [10,11]. Together, these findings indicate that both underdosing and overdosing are clinically harmful and emphasize the need for accurate, guideline-concordant anticoagulant dosing supported by system-level interventions.

Injectable anticoagulants also present important safety challenges. In particular, low-molecular-weight heparin (LMWH) is prone to prescribing errors because dosing depends on body weight, renal function, and indication. Previous studies have reported medication errors in up to 34% of LMWH prescriptions, with most errors attributable to inappropriate dose adjustments based on body weight or renal function [12]. Given LMWH’s narrow therapeutic window, such errors may lead to treatment failure from underdosing or major bleeding from overdosing, reinforcing the need for structured safeguards to support safe prescribing and monitoring.

### Prior Work

Clinical decision support systems (CDSSs) have therefore emerged as key tools to reduce prescribing errors. By integrating clinical information such as diagnoses, laboratory results, and concomitant medications, CDSSs can assess prescription appropriateness and support individualized decision-making, and they have been shown to reduce medication errors and adverse drug events in diverse health care settings [13-21]. Broadly, CDSSs for medication safety can be categorized into rule-based and AI-based approaches, each offering distinct advantages and limitations.

Rule-based CDSSs can reduce prescribing errors by generating targeted, actionable alerts. However, they rely on predefined rules that require continuous curation and maintenance and often fail to capture complex, multifactorial patient contexts that contribute to prescribing errors [22]. Moreover, rule-based systems may generate a high volume of alerts with limited clinical value, contributing to alert fatigue. In South Korea, alerts from the national Drug Utilization Review (DUR) system in tertiary care hospitals have shown an alert override rate of 73%, largely attributed to alert fatigue [23]. Similar studies in other countries have reported override rates ranging from 62% to 95% [24]. Excessive, low-value alerts increase clinician workload and erode trust in CDSSs, underscoring the need to improve alert specificity, prioritization, and clinical relevance.

Machine learning–based CDSSs can provide flexible, data-driven predictions by leveraging complex patterns in large-scale clinical data. Nonetheless, their limited interpretability can impede clinical acceptance, workflow integration, and governance. To bridge the gap between transparency and predictive performance, hybrid CDSSs that combine rule-based logic with machine learning models have recently been proposed. These systems combine the complementary strengths of rule-based and data-driven approaches, enabling reliable detection of well-defined prescribing errors while extending predictive capability to complex clinical contexts not easily captured by fixed rules. For example, a French study demonstrated that a hybrid approach improved both accuracy and reliability in detecting prescribing errors [19].

### Study Objectives

Despite their potential, few hybrid CDSSs have been developed and evaluated in real-world clinical practice, particularly for high-alert medications such as anticoagulants. To mitigate the risks associated with the complex dosing and monitoring requirements of these agents, we developed a hybrid CDSS to detect and prevent prescribing errors involving anticoagulant therapy. The system integrates rule-based logic with a machine learning model and was implemented in 3 tertiary care hospitals for development, internal validation, and external validation to assess performance and clinical utility. We aimed to evaluate whether the hybrid CDSSs could comprehensively identify anticoagulant prescriptions requiring pharmacist intervention.

## Methods

### Study Setting

We conducted a retrospective study using electronic health record (EHR) data from hospital A, a tertiary academic medical center in South Korea with 1616 beds. Hospital A served as the development site for model development, pilot testing, and internal validation. External validation was conducted at 2 tertiary academic medical centers in South Korea, including hospital B (1245 beds) and hospital C (800 beds). The hybrid CDSS was designed to support pharmacist-led prescription review by identifying orders that may require pharmacist intervention. Reporting of this evaluation study followed the STARE-HI (Statement on Reporting of Evaluation Studies in Health Informatics) guideline, and a completed checklist is provided in Table S1 of Multimedia Appendix 1.

### Ethical Considerations

The study was approved by the Institutional Review Board (IRB) of Seoul National University Hospital (IRB number 2307-105-1449). Informed consent was waived due to the use of deidentified retrospective data and the minimal risk posed to patients. Each participating institution obtained separate ethical approval for the external validation (IRB number B-2404-894-403 and IRB number 2024-01-031).

### Hybrid CDSS Development

#### Data Collection

We identified hospitalized patients who had at least one prescription for an oral anticoagulant or LMWH between January 1, 2020, and December 31, 2020. Anticoagulants of interest were apixaban, dabigatran, edoxaban, rivaroxaban, warfarin, and LMWH agents (dalteparin, enoxaparin, and nadroparin).

The EHR dataset comprised patient demographics, diagnoses, laboratory results, concomitant medications, and prescription characteristics (dosing, frequency, route, total daily dose, and prescribing departments). Each anticoagulant order constituted a single observation.

#### Knowledge-Based Component

The knowledge-based component consisted of explicit rules derived from the Korean Ministry of Food and Drug Safety (MFDS), approved prescribing information, and major drug information database [25]. Rules evaluated eligibility, contraindications, dose adjustment, and treatment duration based on patient-specific factors, including indication, age, body weight, renal function, and hepatic function. These factors were selected because they are specified in Korean anticoagulant labeling and available as structured real-time data, whereas factors not routinely available in this form, such as pharmacogenomic status, were not included. Because the recommended dose of DOACs varies by indication, each order was mapped to a presumptive indication using a predefined diagnosis-informed and context-informed hierarchy, allowing the application of indication-specific dosing rules. To minimize misclassification, the hierarchy prioritized indications with more specific temporal or contextual evidence before assigning residual orders to atrial fibrillation. DOAC orders were assigned to VTE treatment when a documented acute VTE diagnosis was present within a prespecified lookback period. If no such diagnosis was identified, orders were classified as postoperative VTE prophylaxis when issued by the orthopedics service in the perioperative setting. Remaining orders were mapped to atrial fibrillation. We evaluated the presumptive indication-mapping hierarchy by comparing automated indication assignments with pharmacist-documented indications obtained through chart review at Seoul National University Hospital. Because dosing logic differs across anticoagulant classes, LMWH dosing rules were primarily stratified by body weight, whereas warfarin dosing was guided by INR monitoring and dose titration rather than indication-specific dosing. The rule engine evaluated label-based eligibility and contraindications and assessed dosing appropriateness, including single dose, dosing frequency, maximum daily dose, and recommended treatment duration, using patient-specific factors. A separate rule set evaluated clinically significant drug-drug interactions based on MFDS labeling, Lexicomp, and Micromedex criteria [25-27].

#### Machine Learning Component

In parallel, we developed a machine learning model to predict whether an anticoagulant order would require pharmacist intervention. At the study hospital, all medication orders undergo prospective pharmacist review. Orders requiring pharmacist intervention are documented with structured reasons. For this study, a prescribing error was defined as an anticoagulant order requiring pharmacist intervention due to dosing or administration unit errors, therapeutic duplication, or safety-related discontinuation. We used the CatBoost classifier (version 1.2.5; Yandex), incorporating demographics, diagnoses, care setting, dosing characteristics, and laboratory values closest to the prescription time.

The 2020 development dataset was randomly split at the order level into a model-training subset (70%) and a model-tuning subset (30%). The split was not performed at the patient level; therefore, prescriptions from the same patient could have appeared in more than one development subset. Hyperparameters were optimized using Bayesian optimization within an automated machine learning framework, with accuracy as the primary objective function. Missing values were managed using the intrinsic mechanism of CatBoost.

#### Integration of Rule-Based and Machine Learning Components

Within the hybrid architecture, each prescription was independently evaluated by the knowledge-based and machine learning components. The knowledge-based component generated alerts based on predefined criteria, while the machine learning component generated the predicted probability that the order would require pharmacist intervention. These outputs were integrated into a single continuous hybrid risk probability. Prescriptions flagged by the knowledge-based component were assigned a probability of 1, whereas all other prescriptions were assigned the machine learning–predicted probability. For each alert, the triggering component and relevant rule violation or predicted risk probability were recorded to support pharmacists’ interpretation.

#### System Implementation and Workflow Integration

Prescription data were retrieved from the EHR server at hourly intervals, and flagged orders were displayed on a web-based dashboard used by pharmacists during routine prospective order review. The dashboard summarized patient characteristics, alert type, and the rationale for each alert.

### Model Evaluation

Pharmacists assessed alert appropriateness across 3 domains: technical accuracy, clinical relevance, and clinical usefulness. Before the evaluation, reviewing pharmacists from all sites participated in a training session to harmonize adjudication criteria across institutions. During the session, representative alert cases and the rationale for each adjudication decision were shared and discussed. All alerts were independently reviewed by 2 pharmacists, and any disagreements were resolved through discussion to reach a consensus. Technical accuracy reflected whether an alert was triggered as intended by the underlying rule logic or model specification when applied to the patient’s actual clinical data at the time of prescribing. An alert was classified as technically correct if the triggering criteria were correctly satisfied and the alert rationale matched the corresponding clinical parameters documented in the record. Clinical relevance captured whether an alert represented a potentially meaningful safety concern in the context of the hospital’s routine prescribing practices and local clinical protocols. Alerts were classified as clinically relevant when pharmacist review was warranted to confirm appropriateness, address a plausible medication safety issue, or rule out an exception. Alerts were classified as irrelevant when, despite meeting technical criteria, they did not require further evaluation due to clear contextual justification or lack of clinical significance in the local setting. Clinical usefulness evaluated the downstream value of the alert in supporting pharmacists’ decision-making and workflow. Alerts were classified as useful if they prompted an in-depth review of the medical record or prescription details and contributed to an actionable decision, including contacting the prescriber, recommending changes, or documenting the rationale for continuing therapy after a structured review. Alerts were classified as not useful if they were acknowledged without meaningful assessment, did not change the level of review beyond usual practice, or did not inform any clinical decision.

Additionally, all anticoagulant prescriptions were independently reviewed by at least 2 pharmacists to establish reference labels for model performance evaluation. Model discrimination was assessed using the area under the receiver operating characteristic curve (AUROC) and the area under the precision-recall curve (AUPRC), with additional metrics, including accuracy, sensitivity, specificity, positive predictive value, and negative predictive value. CIs were estimated using the DeLong method for AUROC and exact Clopper-Pearson intervals for proportion-based metrics. The CIs for the AUPRC were obtained by bootstrap resampling with 1000 replications.

### Pilot Testing and Validation

The 2 rounds of pilot testing were conducted from November 23, 2023, to November 29, 2023 (excluding weekend days), and on December 8, 2023, respectively. These rounds were conducted to identify technical issues and refine rules prior to deployment. Prescriptions reviewed during the pilot phase were subsequently combined with the complete 2020 development dataset to update the machine learning component of the CDSS, resulting in the final model for deployment. These pilot-tested prescriptions were not included in any of the reported validation datasets. After the application of the finalized CDSS model, internal validation was conducted at hospital A through pharmacist review of all anticoagulant prescriptions issued on 2 predefined dates in 2024: March 26 and April 29.

External validation was conducted after the CDSS had been finalized. Local preprocessing at each external institution was limited to mapping EHR data to the finalized CDSS input structure and did not involve adjudication labels, model retraining, threshold selection, site-specific calibration, or performance optimization. Pharmacist adjudication was conducted independently according to prespecified criteria. External validation was conducted at 2 tertiary hospitals: hospital B and hospital C. Retrospective validation was performed at both sites during routine clinical practice from September 23, 2024, to September 27, 2024, and an additional prospective validation was conducted at hospital C on December 23, 2024.

## Results

### Study Setting and Data Collection

For model development, we extracted 75,200 anticoagulant prescriptions from hospital A in 2020, of which 812 (1.1%) were classified as prescribing errors. During 2 pilot testing periods, an additional 435 prescriptions were reviewed, and 9 prescribing errors were identified; these data were incorporated into the training dataset for model updating.

For internal validation, 466 anticoagulant prescriptions issued on March 26 and April 29, 2024, were reviewed, with 12 (2.6%) prescriptions adjudicated as prescribing errors. Among 284 DOAC prescriptions in the internal validation set, the automated indication mapping agreed with the documented indication for 252 (88.7%) prescriptions (Table S2 in Multimedia Appendix 1). External validation was conducted at 2 tertiary hospitals. At hospital B, 966 prescriptions were reviewed over 5 days in September 2024, and 82 (8.5%) prescribing errors were identified. At hospital C, 417 and 31 prescriptions were reviewed during the first and second validation periods, yielding 22 (5.3%) and 4 (12.9%) prescribing errors, respectively. The proportions of missing values for each hospital are presented in Table S3 in Multimedia Appendix 1.

### Hybrid CDSS Development and Refinement

The hybrid CDSS integrated a rule-based system with a machine learning model. The initial rule set comprised 38 patient-specific rules addressing diagnosis, body weight, age, renal function, and hepatic function, as well as 1129 rules for contraindicated drug-drug interactions involving anticoagulants.

**Figure 1.**
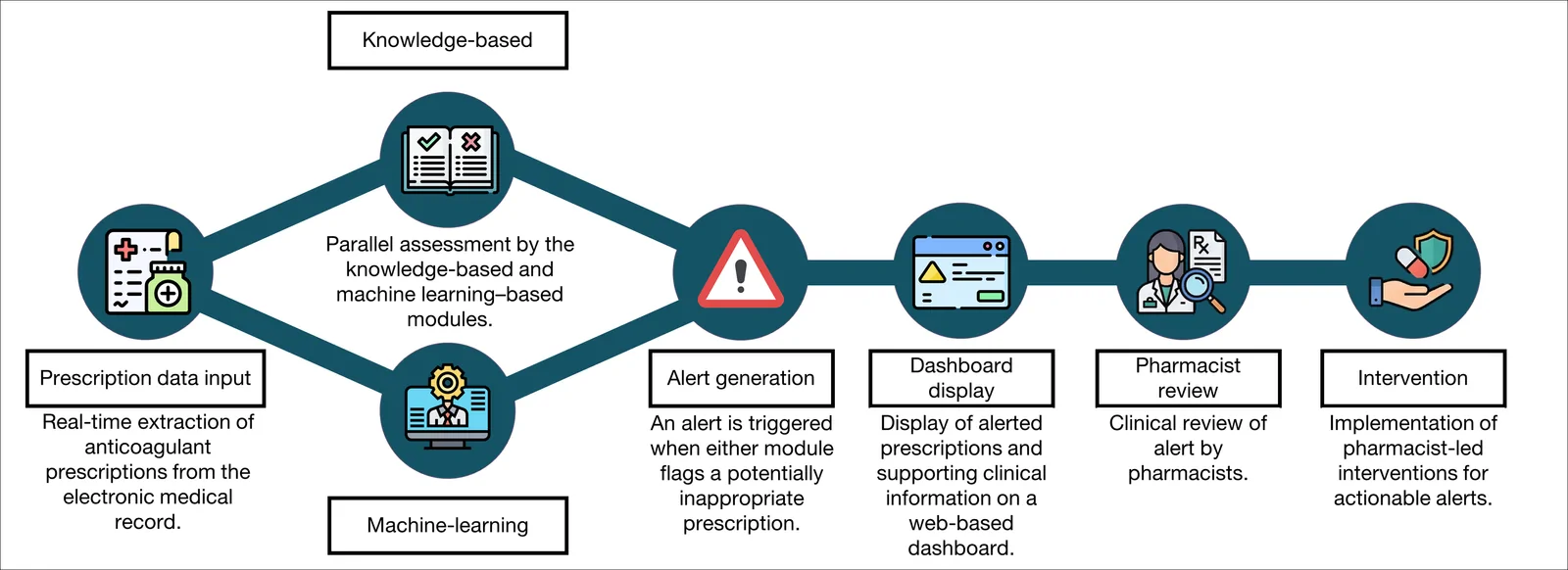
Workflow of a hybrid clinical decision support system.

Across 2 pilot-testing rounds, 667 alerts were generated, of which 583 (87.4%) were judged technically correct. Pilot testing identified several technical issues, including the retrieval of outdated laboratory results and incorrect treatment duration calculations. As a result, 9 existing rules were revised and 6 new rules were added. Prescriptions reviewed during the pilot phase were subsequently incorporated into the training dataset to update the machine learning model. The workflow of the hybrid CDSS is presented in Figure 1, with the system architecture and dashboard screenshots provided in Figures S1 and S2 of Multimedia Appendix 1, respectively.

### Internal and External Validation

During internal validation at hospital A, the hybrid CDSS generated 88 alerts, corresponding to an alert rate of 18.9% (88/466). All alerts were technically correct. Of these, 78 (88.6%) alerts were judged clinically relevant, 76 (86.4%) were considered clinically useful, and 12 (13.6%) led to pharmacist interventions. Representative examples of alerts are shown in Table S4 of Multimedia Appendix 1.

In external validation across 3 site-periods, alert rates ranged from 22.6% (7/31) to 32.1% (310/966), with the number of alerts per site ranging from 7 to 310. Across sites, 93.2% (109/117) to 100% (7/7) were technically correct, 78.4% (243/310) to 91.5% (107/117) were clinically relevant, 26.5% (82/310) to 85.7% (6/7) were clinically useful, and 18.8% (22/117) to 57.1% (4/7) required pharmacist intervention (Table 1).

**Table 1.** Alert appropriateness in internal and external validation.

Alert evaluation^a^	Internal validation	External validation, retrospective		External validation, prospective
	Hospital A	Hospital B	Hospital C (first)	Hospital C (second)
Overall prescriptions, n	466	966	417	31
Total alerts, n (%)	88 (18.9)	310 (32.1)	117 (28.1)	7 (22.6)
Technically correct alerts	88 (100)	309 (99.7)	109 (93.2)	7 (100)
Clinically relevant alerts	78 (88.6)	243 (78.4)	107 (91.5)	6 (85.7)
Clinically useful alerts	76 (86.4)	82 (26.5)	77 (65.8)	6 (85.7)
Pharmacist intervention	12 (13.6)	82 (26.5)	22 (18.8)	4 (57.1)

a Values are presented as n (%). Percentages for total alerts are calculated as the proportion of overall prescriptions at each site; percentages for alert appropriateness and pharmacist intervention are calculated as the proportion of total alerts at each site. Hospital C (second) represents the prospective external validation.

Among rule-based alerts, drug-drug interactions were the most frequently identified category, accounting for 28.6% (2/7) to 47.9% (56/117) of alerts, followed by contraindications and overdosing. The distribution of alert categories varied across institutions (Table 2).

**Table 2.** Distribution of alert categories across internal and external validation.

Alert evaluation	Internal validation	External validation, retrospective		External validation, prospective
	Hospital A	Hospital B	Hospital C (first)	Hospital C (second)^a^
Total alerts, n	88	310	117	7
Rule-based alerts, n (%)	67 (76.1)	199 (64.2)	108 (92.3)	7 (100)
Drug interaction	38 (43.2)	114 (36.8)	56 (47.9)	2 (28.6)
Contraindication	17 (19.3)	60 (19.4)	27 (23.1)	1 (14.3)
Overdosing	10 (11.4)	49 (15.8)	31 (26.5)	4 (57.1)
Dosing frequency	2 (2.3)	5 (1.6)	1 (0.9)	0 (0)
Wrong indication	1 (1.1)	2 (0.6)	17 (14.5)	1 (14.3)
Machine learning–based alerts, n (%)	23 (26.1)	122 (39.4)	10 (8.5)	1 (14.3)

a All percentages are calculated as a proportion of total alerts at each site. Categories are not mutually exclusive; a single prescription may trigger multiple rule violations, and rule-based and machine learning–based components may both flag the same prescription. Category and component counts may therefore sum to more than the total number of alerts.

No false negatives were observed for prescriptions requiring pharmacist intervention in the internal or external validation datasets. The AUROC of the hybrid CDSS was 0.916 (95% CI 0.899‐0.933) in internal validation, ranged from 0.871 to 0.880 in the 2 retrospective external validations, and was 0.963 in the small prospective feasibility validation. The AUPRC ranged from 0.568 to 0.833 across validation sites, exceeding the corresponding baseline prevalence at every site (Table 3).

**Table 3.** Clinical decision support systems (CDSSs) performance in internal and external validation.

Alert evaluation	AUROC^a^, PE^b^ (95% CI)	AUPRC^c^, PE (95% CI)	Accuracy, PE (95% CI)	Specificity, PE (95% CI)	Sensitivity, PE (95% CI)	PPV^d^, PE (95% CI)	NPV^e^, PE (95% CI)
Internal validation							
Hospital A	0.916 (0.899‐0.933)	0.568 (0.534‐0.607)	0.837 (0.800‐0.869)	0.833 (0.795‐0.866)	1.000 (0.735‐1.000)	0.136 (0.072‐0.226)	1.000 (0.990‐1.000)
External validation, retrospective	0.873 (0.861‐0.885)	0.624 (0.604‐0.646)	0.766 (0.743‐0.789)	0.747 (0.723‐0.771)	1.000 (0.965‐1.000)	0.244 (0.204‐0.287)	1.000 (0.996‐1.000)
Hospital B	0.871 (0.856‐0.885)	0.632 (0.607‐0.656)	0.764 (0.736‐0.790)	0.742 (0.712‐0.771)	1.000 (0.956‐1.000)	0.265 (0.216‐0.317)	1.000 (0.994‐1.000)
Hospital C (first)	0.880 (0.859‐0.901)	0.594 (0.560‐0.629)	0.772 (0.729‐0.812)	0.759 (0.714‐0.801)	1.000 (0.846‐1.000)	0.188 (0.122‐0.271)	1.000 (0.988‐1.000)
External validation, prospective							
Hospital C (second)	0.963 (0.913‐1.000)	0.833 (0.600‐1.000)	0.903 (0.742‐0.980)	0.889 (0.708‐0.976)	1.000 (0.398‐1.000)	0.571 (0.184‐0.901)	1.000 (0.858‐1.000)

a AUROC: area under the receiver operating characteristic curve.

b PE: point estimates.

c AUPRC: area under the precision-recall curve.

d PPV: positive predictive value.

e NPV: negative predictive value.

## Discussion

### Principal Findings

In this multicenter study, we developed and externally validated a hybrid CDSS that integrates a rule-based model and a machine learning model to prevent prescription errors involving anticoagulants. Using pharmacist-adjudicated interventions as the reference standard, the system demonstrated high discriminative performance, with AUROC values of 0.916 in internal validation and 0.871 to 0.963 across external validation sites. Moreover, alerts generated by the system were consistently judged to be technically correct, clinically relevant, and clinically useful. Together, these findings suggest that a hybrid CDSS can enhance prescription review for high-risk medications while maintaining a clinically acceptable alert profile across hospitals with heterogeneous practice environments.

### Comparison With Prior Work

Medication errors involving anticoagulants are common and clinically important. Among various types of errors, inappropriate dosing remains the most frequent and critical concern [2]. Systematic reviews have reported that 13% to 37% of DOAC prescriptions in real-world practice are inappropriately dosed, often because patient-specific factors such as age, body weight, and renal function are not adequately considered [2]. Furthermore, Ko et al [28] identified impaired renal function, advanced age, and prior DOAC exposure as key risk factors for inappropriate dosing. These findings underscore that patients on long-term anticoagulation may be particularly vulnerable to errors, as their clinical parameters, such as renal function and weight, evolve over time. In our study, the proportion of prescriptions classified as prescribing errors varied across institutions and study periods, from 1.1% (812/75,200) in the development set, based on routine pharmacist interventions, to 2.6% (12/466) to 12.9% (4/31) in validation samples. This heterogeneity likely reflects not only true differences in prescribing patterns and patient risk but also differences in review intensity, local workflows, and how intervention-worthy problems are operationalized by pharmacists. Importantly, despite this variability, the hybrid CDSS consistently identified all prescriptions requiring pharmacist intervention, suggesting that it can function as a reliable safety net across diverse clinical settings.

Although DOACs are often perceived as safer and simpler than warfarin because of their fixed dosing, their dose adjustment criteria remain complex and difficult to fully implement within conventional DUR systems. Consequently, off-label or suboptimal dosing is frequently observed in clinical practice. To address these challenges, our hybrid CDSS encoded eligibility, contraindication, and dose-adjustment rules derived from product labeling and major drug information databases, while also incorporating laboratory data and concomitant medication information. By combining explicit rule-based logic with a machine learning model trained on historical pharmacist interventions, the system was able to capture broader aspects of patient context, including patterns that are difficult to formalize as static rules alone.

Across internal and external validation, the hybrid CDSS generated alerts for 18.9% (88/466) to 32.1% (310/966) of anticoagulant prescriptions. Notably, no false-negative cases were observed in the validation sample, suggesting high sensitivity for identifying prescriptions requiring pharmacist intervention within the evaluated periods. Prior CDSS studies illustrate the trade-off between alert burden and safety coverage. For example, MedGuard reported alerts for only 2.36% of prescriptions [13], but did not assess the risk of missed high-risk cases. Conversely, systems such as Check of Medication Appropriateness (CMA) and PharmaCheck produced high alert volumes, with a limited proportion of alerts judged clinically relevant [15,21]. In this context, the hybrid CDSS showed a potentially useful balance between alert burden and safety coverage. This balance was achieved with a moderate alert rate, and no false-negative cases were observed in the validation samples. However, the absence of false negatives should be interpreted cautiously. This finding may partly reflect the short validation periods and the limited number of prescriptions requiring intervention. Further evaluation in larger and longer-term cohorts is needed to determine whether this apparent safety coverage can be maintained in routine anticoagulant prescribing.

In internal and external validations, 26.5% (82/310) to 86.4% (76/88) of alerts were judged clinically useful, and 13.6% (12/88) to 57.1% (4/7) led to pharmacist intervention. These findings indicate that the system not only identified prescriptions warranting review but also captured a substantial proportion of cases in which active intervention was required to ensure patient safety. Although few studies have reported comparable metrics specifically for anticoagulants, prior pharmacist-oriented CDSS evaluations provide relevant context. In PharmaCheck, 20.1% of alerts resulted in pharmacist intervention, whereas only 1.6% did so in the CMA [15,21]. Additionally, the AUROC values observed in our study compare favorably with those reported for a previously reported hybrid CDSS (AUROC 0.81) [19]. While direct comparisons are limited by differences in target medications and outcomes, the relatively high proportion of alerts leading to intervention in our study suggests that the developed hybrid CDSS represents a competitive and clinically impactful approach.

In this study, we implemented a hybrid CDSS that integrates rule-based logic with machine learning models. The system provides explicit rule-based alerts for prescriptions contraindicated by renal function or involving clinically significant drug-drug interactions, while also generating risk-based alerts for marginal dosing patterns and other clinically ambiguous situations not readily captured by predefined rules. This design supports both clearly defined safety checks and broader identification of clinically relevant prescribing risks.

Another important feature of this system is its pharmacist-centered design. Rather than delivering alerts directly to physicians, the CDSS was designed to support pharmacists as primary users who triage, interpret, and contextualize alerts before escalation. Prior work has shown that pharmacist-mediated filtering can substantially increase the positive predictive value of alerts, from 20.1% to 71% in one study of PharmaCheck [15]. In contrast to physician-facing CDSS that often contribute to alert fatigue, our system presents alerts to pharmacists via a dashboard, enabling efficient prioritization of high-risk prescriptions and minimizing unnecessary interruptions to prescribers. In this context, the relatively low positive predictive values of 0.136 to 0.571 can be understood as a consequence of prioritizing comprehensive detection, while the overall alert rate of 18.9% (88/466) to 32.1% (310/966) remains comparable to those reported for other CDSSs. Importantly, this review burden is handled within the established pharmacist triage process rather than being added directly to the prescriber workflow. This workflow design aligns decision support with existing medication review processes and enhances both efficiency and acceptability.

### Limitations

Several limitations should be considered. First, both internal and external validation periods were relatively short, ranging from 1 to 5 days. The reference standard required exhaustive prescription-by-prescription chart review by 2 pharmacists for each validation day. Therefore, validation was deliberately limited to predefined index days to ensure complete manual adjudication. These short validation periods may not fully capture long-term operational challenges such as changes in prescription patterns, system drift, or evolving user behavior. Longer-term prospective evaluations are needed to assess durability and support continuous refinement. In addition, the absence of false negatives in the validation samples should be interpreted cautiously, given the short validation periods, small number of intervention-requiring prescriptions, wide CIs, and the permissive hybrid structure in which alerts were generated when either component flagged an order.

Second, the development dataset was split at the order level rather than the patient level. Therefore, prescriptions from the same patient could have appeared in more than one development subset, which may have led to optimistic development-stage performance estimates. However, the reported temporal and external validation datasets were independent of the development split. Third, the higher proportion of machine learning–based alerts observed at one external site was likely attributable to greater missingness in laboratory data. Alerts triggered by missing information may contribute to alert fatigue if not clearly distinguished from alerts based on abnormal values; however, missingness may also carry clinically meaningful information. For example, missing hematologic or laboratory values may indicate insufficient monitoring in patients receiving anticoagulants, and such alerts may therefore provide a monitoring-related safety message. These findings highlight the importance of explicit alert categorization and complementary strategies to improve data completeness. In addition, alerts related to drug-drug interactions accounted for 29% to 48% of all alerts, yet only a limited proportion resulted in pharmacist intervention. This discrepancy suggests that the frequency of interaction-related alerts may exceed their clinical relevance and contribute to alert fatigue. Stratifying interaction alerts into contraindicated and precautionary categories may improve clinical utility and reduce unnecessary interruptions. Fourth, the system was developed specifically for anticoagulants, limiting its immediate applicability to other drug classes. Nevertheless, successful implementation in this high-risk domain suggests that the underlying framework may be extendable to other high-alert medications with appropriate adaptation. Fifth, reliance on structured data precluded the use of unstructured clinical information such as free-text notes or surgical reports. Although surrogate indicators were used, future work incorporating natural language processing may enhance contextual awareness. Pharmacogenomic determinants of warfarin dosing, such as *CYP2C9* and *VKORC1* polymorphisms, were also not incorporated, reflecting routine clinical practice in which warfarin is generally initiated at a standard dose and adjusted according to INR monitoring rather than guided by pre-emptive genotyping. Incorporating pharmacogenomic information may further refine the knowledge base in the future. Additionally, we performed both internal and external validation in tertiary care hospitals, and further studies are needed to assess the applicability of the model in other health care settings. Finally, the reference standard was based on pharmacist-documented interventions, which may underestimate the true error rate by missing unrecognized or undocumented errors and introduce label noise. The machine learning component was trained using pharmacist-documented interventions from a single tertiary center with established pharmacist review practices, so the resulting labels may partly reflect a site-specific intervention standard. Because pharmacist intervention practices vary across institutions, the model’s alerts and performance may generalize less well, and site-specific recalibration may be needed before implementation in new settings. More sophisticated integration strategies, such as risk-tiered alerting or dynamic weighting of rule-based and model-based signals, may further improve performance and warrant future investigation. Also, the model was not optimized for precision-oriented metrics, which may leave an unnecessary pharmacist review burden. Future refinement could improve precision while maintaining adequate safety coverage. This study did not evaluate clinical outcomes, including bleeding, thromboembolism, adverse drug events, length of stay, mortality, or actual reductions in prescribing errors. Accordingly, the findings should be interpreted as evidence of the system’s ability to identify prescriptions requiring pharmacist review, and future prospective studies should determine whether this translates into measurable improvements in patient safety.

### Conclusions

In conclusion, we developed a hybrid CDSS integrating rule-based logic with machine learning to detect prescribing errors involving anticoagulants and, for the first time in Korea, evaluated it through multicenter external validation. The system demonstrated robust performance, high clinical relevance and usefulness, and a substantial proportion of alerts leading to pharmacist intervention, without missing prescriptions requiring intervention. By centering pharmacists in the alert workflow, the system minimizes unnecessary alerts to physicians while supporting efficient prioritization of high-risk prescriptions. These findings suggest that the hybrid CDSS is a practical and scalable tool for supporting pharmacist-led review and pharmacist workflow efficiency in the management of high-risk medications.

## Supplementary material

10.2196/101202 Multimedia Appendix 1 Clinical decision support system development, validation data, system architecture, and alert evaluation.

## Abbreviations

AUPRC: area under the precision-recall curve
AUROC: area under the receiver operating characteristic curve
CDSS: clinical decision support system
CMA: Check of Medication Appropriateness
DOAC: direct oral anticoagulant
DUR: Drug Utilization Review
EHR: electronic health record
INR: international normalized ratio
IRB: institutional review board
LMWH: low-molecular-weight heparin
MFDS: Ministry of Food and Drug Safety
STARE-HI: Statement on Reporting of Evaluation Studies in Health Informatics
VTE: venous thromboembolism
