# Supplementary material for: A Hybrid Rule-Based and Machine Learning–Based Clinical Decision Support System to Support Prescription Review: Development and External Validation Study
Source: J Med Internet Res. 2026 Sep 23;28:e101202. doi: 10.2196/101202 (PMC13600658; doi:10.2196/101202)
Supplement: Supplementary file 1 [file jmir-v28-e101202-s001.docx]

**Supplemental materials**

**A Hybrid Rule-Based and Machine Learning Clinical Decision Support System to Support Prescription Review: Development and External Validation**

**Authors and affiliations**

Jonghyun Jeong^1*^, Kyu-Nam Heo^1,2*^, A Jeong Kim^3^, Jin Hee Baek^3^, Yun Hee Jo^3^, Sunghwan Kim^3^, Mingi Jo^4^, Young-Mi Ah^5^, Ji Min Han^6^, Sae Won Choi^7^, Junho Song^4^, Sangil Min^8^, and Ju-Yeun Lee^1,2^

^1^College of Pharmacy, Seoul National University, Seoul, Republic of Korea

^2^College of Pharmacy and Research Institute of Pharmaceutical Sciences, Seoul National University, Seoul, Republic of Korea

^3^Department of Pharmacy, Seoul National University Hospital, Seoul, Korea

^4^ZerOne AI Inc, Seoul, Republic of Korea

^5^College of Pharmacy, Yeungnam University, Gyeongsangbuk-do, Republic of Korea

^6^College of Pharmacy, Chungbuk National University, Cheongju, Republic of Korea

^7^Office of Hospital Information, Seoul National University Hospital, Seoul, Republic of Korea

^8^Department of Surgery, Seoul National University Hospital, Seoul, Korea

**Supplementary materials index**

| Supplementary Table S1. STARE-HI Checklist  Supplementary Table S2. Agreement between automated indication mapping and documented indications in direct oral anticoagulants  Supplementary Table S3. Proportion of missing values across validation sites  Supplementary Figure S1. System architecture of the clinical decision support system  Supplementary Figure S2. Visualization of alerts in the hybrid CDSS dashboard  Supplementary Table S4. Examples of alert categories identified during internal/external validation | Page 1  Page 1  Page 2  Page 3  Page 4  Page 5 |
| --- | --- |

Supplementary Table S1. STARE-HI Checklist

| **STARE-HI Item** | **Item on page** |
| --- | --- |
| **Title** | 1 |
| **Abstract** | 3 |
| **Keywords** | 4 |
| **Introduction** | 5 - 7 |
| Scientific background | 5 - 6 |
| Rationale for the study | 6 - 7 |
| Objectives of the study | 7 |
| **Study context** | 8 |
| Organizational setting | 8, Supplementary Figure 1 |
| System details and system in use | 8, Supplementary Figure 1 |
| **Methods** | 8 - 12 |
| Study design | 8 |
| Theoretical background | 9 - 11 |
| Participants | 11 - 12 |
| Study flow | 8 - 12, Figure 1 |
| Outcome measure or evaluation criteria | 10 - 11 |
| Methods for data acquisition and measurement | 11 - 12 |
| Methods for data analysis | 11 - 12 |
| **Results** | 12 - 18 |
| Demographic and other study coverage data | 12, Table 1 |
| Unexpected events during the study | NA |
| Study findings and outcome data | 12 - 18 |
| Unexpected observations | 13 |
| **Discussion** | 19 - 23 |
| Answers to study questions | 19 |
| Strengths and weaknesses of the study | 19 - 21 |
| Results in relation to other studies | 19 - 21 |
| Meaning and generalizability of the study | 21 - 22 |
| Unanswered and new questions | 22 - 23 |
| **Conclusion** | 23 |
| **Authors’ contribution** | 24 - 25 |
| **References** | 27 - 30 |
| **Appendices** | Supplementary materials |

Supplementary Table S2. Agreement between automated indication mapping and documented indications in direct oral anticoagulants

|  | |  | **Documented indication** | | | |
| --- | --- | --- | --- | --- | --- | --- |
|  |  | **Atrial fibrillation** | **VTE treatment** | **VTE prophylaxis** | **Others** | **Total** |
| **Automated indication mapping** | **Atrial fibrillation** | 213 | 11 | 0 | 15 | 239 |
|  | **VTE treatment** | 0 | 36 | 0 | 1 | 37 |
|  | **VTE prophylaxis** | 4 | 1 | 3 | 0 | 8 |
|  | **Total** | 217 | 48 | 3 | 16 | 284 |

Supplementary Table S3. Proportion of missing values across validation sites

| **Variables** | **Hospital A** | **Hospital B** | **Hospital C 1^st^** | **Hospital C 2^nd^** |
| --- | --- | --- | --- | --- |
| Sex, male | 0 (0) | 0 (0) | 0 (0) | 0 (0) |
| Age, years | 0 (0) | 0 (0) | 0 (0) | 0 (0) |
| Weight, kg | 0 (0) | 0 (0) | 0 (0) | 0 (0) |
| SCr, mg/dL | 42 (9.0) | 235 (24.3) | 62 (14.9) | 2 (6.5) |
| CrCl, mL/min | 61 (13.1) | 235 (24.3) | 112 (26.9) | 2 (6.5) |
| Hb, g/dL | 42 (9.0) | 288 (29.8) | 115 (27.6) | 6 (19.4) |
| PLT, x10^3^/uL | 42 (9.0) | 288 (29.8) | 62 (14.9) | 2 (6.5) |
| INR | 64 (13.7) | 429 (44.4) | 79 (18.9) | 3 (9.7) |
| D-dimer | 228 (48.9) | 718 (74.3) | 178 (42.7) | 10 (32.3) |
| AST, IU/L | 40 (8.6) | 355 (36.7) | 62 (14.9) | 2 (6.5) |
| ALT, IU/L | 40 (8.6) | 355 (36.7) | 62 (14.9) | 2 (6.5) |
| Tb, mg/dL | 40 (8.6) | 355 (36.7) | 62 (14.9) | 2 (6.5) |

Supplementary Figure S1. System architecture of the clinical decision support system

Supplementary Figure S2. Visualization of alerts in the hybrid CDSS dashboard

Supplementary Table S4. Examples of alert categories identified during internal/external validation

| **Category** | **Example** |
| --- | --- |
| Technically incorrect | Retrieval of outdated laboratory values that generated an incorrect alert |
| Technically correct | Alert generated using accurate and up-to-date patient information. |
| Clinically irrelevant | An AI alert was generated despite being an appropriate prescription when considering body weight and renal function |
| Clinically relevant | Alert was generated for a patient with a creatinine clearance of 14 mL/min/1.73 m², indicating a clinically significant dosing risk that required pharmacist review. |
| Clinically uselessness | Alert generated in outpatients due to missing renal function data within the most recent 3 months  Alert triggered during inpatient care where the concomitant use of clopidogrel, aspirin, or NSAIDs was clinically intentional |
| Clinically useful | Alert was generated due to an underdose prescription when considering body weight and renal function  Alert was generated due to an overdose prescription when considering body weight and renal function  Alert was triggered by prolonged use of high-dose VTE initial treatment beyond the recommended duration |
